# “Ozempic Face” in Plastic Surgery: A Systematic Review of the Literature on GLP-1 Receptor Agonist Mediated Weight Loss and Analysis of Public Perceptions

**DOI:** 10.1093/asjof/ojaf056

**Published:** 2025-06-11

**Authors:** Giulia Daneshgaran, Orr Shauly, Daniel J Gould

## Abstract

**Background:**

Glucagon-like peptide-1 (GLP-1) receptor agonists have gained popularity after clinical trials demonstrated their weight-loss potential. The term “Ozempic® face” has been coined to describe the exaggerated volume loss from semaglutide therapy, resulting in advanced facial aging.

**Objectives:**

The authors of this study aim to review plastic surgery publications discussing GLP-1 receptor agonists and characterize the public's perception of the effect of these medications on weight loss.

**Methods:**

A systematic review of the PubMed database was conducted to identify articles discussing GLP-1 receptor agonist use in plastic surgery. Articles in non-English languages or on nonhuman subjects were excluded. Bias assessment was completed using standardized checklists. Google Trends was used to track public interest in these medications and their effect on face and body morphology.

**Results:**

Twenty-three articles were identified, revealing that (1) several injectable drugs are available for weight loss, (2) GLP-1 receptor agonists cause morphological changes resembling advanced aging, (3) surgical and nonsurgical options exist to address these changes, (4) adverse effects of these medications help guide perioperative management, and (5) important contraindications exist to their use. Online searches for “Ozempic® face” were linked to rising searches for “face filler” and “plastic surgeons.”

**Conclusions:**

As the unintended morphological changes of GLP-1 receptor agonists become increasingly reported, so does the public interest in seeking rejuvenation procedures. Although limited by study biases, this systematic review can help the plastic surgery community prepare for the rising needs of this patient population by understanding the risk and benefit profile of GLP-1 receptor agonists and developing clear clinical practice guidelines.

**Level of Evidence: 4 (Therapeutic):**

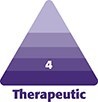

In the United States, the obesity epidemic affects over 100 million adults and almost 15 million children, representing 41.9% of the adult population and 19.7% of the child population.^1,2^ Obesity is defined as having a BMI over 30.0 kg/m^2^ and is linked to many significant chronic diseases, including diabetes, heart disease, and some types of cancer. This epidemic contributes to $173 billion in annual healthcare costs.^1^

Glucagon-like peptide-1 (GLP-1) receptor agonists are a class of medications that stimulate insulin secretion and have become exceedingly popular agents for medication-assisted weight loss, both with on-label and off-label use.^3^ These include semaglutide, which is marketed as Ozempic (Novo Nordisk, Bagsværd, Denmark) and Wegovy (Novo Nordisk), tirzepatide, which is marketed as Mounjaro (Eli Lilly and Company, Indianapolis, IN) and Zepbound (Eli Lilly and Company), and liraglutide, which is marketed as Saxenda (Novo Nordisk). Originally developed to counteract insulin resistance in patients with Type 2 diabetes, GLP-1 receptor agonists are administered subcutaneously with weekly or biweekly dosing.

Recent clinical trials have demonstrated that GLP-1 receptor agonists help with weight loss, lower hemoglobin A1c, and improve cardiovascular health. The Semaglutide Treatment Effect in People with Obesity 1 study found that the weekly administration of semaglutide improved waist circumference, blood pressure, physical function, and overall cardiometabolic health.^4^ Patients using semaglutide for 68 weeks lost 14.9% of their body weight, compared with 2.4% weight loss in those on placebo. In an extension trial, continued use of semaglutide led to a sustained loss of 17.3% of body weight.^5^ Unfortunately, 1 year after stopping the drug, patients regained two-thirds of the weight they had lost, and their cardiometabolic improvements returned to baseline levels. This suggests that long-term use of GLP-1 receptor agonists is likely necessary to maintain results.

Approximately 13% of US adults, accounting for 32 million people, have used a GLP-1 receptor agonist at some point in their lives. About half of those individuals are actively using a prescription for this class of medications.^6^ With the rise in popularity of GLP-1 receptor agonists, a new terminology has been coined by the public and plastic surgery community alike to characterize some of the unintended effects of these medications. Terms like “Ozempic® face,” “Ozempic® butt,” and “Ozempic® body” have become widely recognized to refer to the loss of tissue volume resulting from semaglutide therapy, with plastic surgeons extensively discussing these concepts on social media, podcasts, and blogs.^7-9^ This trend points to a rising group of plastic surgery patients seeking rejuvenation following soft tissue loss as a result of medication-assisted weight loss.

In this study, the authors aim to compile and review the most up-to-date published data on the safety, efficacy, and clinical practice guidelines for the use of GLP-1 receptor agonists in plastic surgery and associated specialties. Along with a systematic review of the literature, a Google Trends analysis (Google LLC, Mountain View, CA) and Google News search is conducted to qualify public perception of GLP-1 receptor agonists and their association with weight loss. This aggregate data will allow plastic surgeons to more accurately counsel and treat their patients who are already utilizing or considering use of these medications.

## METHODS

A systematic review was conducted in accordance with the Preferred Reporting Items for Systematic Reviews and Meta-Analyses (PRISMA) guidelines (available in Supplemental Tables 1, 2). A comprehensive search was conducted using the PubMed database (US National Institutes of Health, Bethesda, MD) between February 15 and February 22, 2025. The keywords used are represented in Supplemental Figure 1 and included a combination of “GLP-1” or “GLP-1 agonist” or “GLP-1 receptor agonist” or “glucagon-like peptide 1” or “glucagon-like peptide 1 agonist” or “glucagon-like peptide 1 receptor agonist” or “semaglutide” or “liraglutide” or “exenatide” or “tirzepatide” or “dulaglutide” or “Ozempic®” or “Wegovy®” or “Mounjaro®” or “Zepbound®” along with “plastic surgery” or “aesthetic surgery” or “cosmetic surgery” or “facial surgery” or “reconstructive surgery.” The resulting articles were evaluated for relevance and included if they explored the use of GLP-1 receptor agonists in plastic surgery, including but not limited to facial aesthetic, breast, and body surgeries. Articles were excluded if they focused on nonhuman subjects, were published in any language other than English, or if their topic was not relevant to the focus of our study. The reference list of identified literature was screened to obtain additional relevant material as well. All remaining articles were subject to a qualitative review of textual evidence with analysis based on commonly occurring themes. The primary data points collected included study details, authors and date of publication, journal of publication, study type, and summary of findings and/or recommendations. A bias assessment was completed utilizing the Modified Downs and Black Checklist for Assessment of Methodological Quality, which was scored out of 28 points for controlled studies, 20 points for uncontrolled studies, and 16 points for cross-sectional studies. The Joanna Briggs Institute Critical Appraisal Checklist was used to conduct a bias assessment of case reports and was scored out of 8 points.^10,11^ Study quality was determined from the score obtained using the bias assessment checklist divided by the total possible points. Studies that scored >90% were rated as “excellent,” >70% as “good,” >50% as “fair,” and ≤50% as “poor.”^10^ Records were screened and data were collected by 1 author (G.D.) then independently reviewed by the remaining co-authors (O.S. and D.J.G.).

A Google Trends and Google News search was conducted to qualify public perceptions on GLP-1 receptor agonists and their association with the morphological changes seen with medication-assisted weight loss. Google Trends and Google News are publicly available online tools useful for analyzing public interest in various topics and staying updated on global news, respectively. Google Trends allows users to track the popularity of specific search terms over time and provides insight into what people are searching on Google. The relative search volume of a keyword in a specific region or country is measured over time and displayed graphically. Each search term is assigned a score between 0 and 100 at predetermined time points, with 100 indicating peak popularity and 0 signifying insufficient search data. The analysis includes a list of terms named “related queries” that are frequently and concurrently searched with the keyword of interest. When a related query is marked as “breakout,” its search growth exceeds 5000%, meaning it is trending and experiencing a major spike in interest. For the purpose of this analysis, the various keywords searched were “semaglutide,” “Ozempic®,” “Wegovy®,” “Mounjaro®,” and “Zepbound®”; “Ozempic® weight loss,” “Ozempic® face,” “Ozempic® butt,” and “Ozempic® body”; and “facial balancing,” “face filler,” and “hyaluronic acid injections.” The search location was “U.S.A.,” the search timeframe was “past 5 years,” and the search date was October 10, 2024. Google Trends results were analyzed by measuring the overall rate of change for each search term over time. A search for these keywords was also conducted on Google News, a news aggregator service that displays articles from a variety of news outlets across the internet, to further qualify the trends observed in the Google Trends analysis.

## RESULTS

### Systematic Review of the Literature

**Figure 1.**
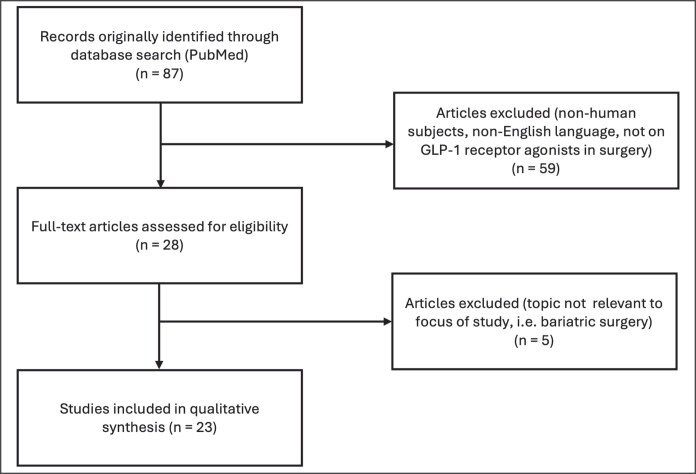
Preferred Reporting Items for Systematic Reviews and Meta-Analyses attrition diagram of literature search for systematic review.

As depicted in the PRISMA diagram (Figure 1), 86 original articles were initially identified through our PubMed search. Of these articles, 58 were excluded based on their language and subject focus. After screening the remaining 28 abstracts, 5 were excluded because their topic was too general or not directly relevant to the focus of our study. No additional literature items were identified from the reference lists of the included articles. Overall, 23 articles were included in the systematic review. The articles were characterized by year of publication, article type, journal of publication, and article focus. As seen in Table 1, 96% of articles were published between the years 2023 and 2025. The article types included: correspondence and communications pieces (n = 5), review articles (n = 5), case reports (n = 4), retrospective cohort studies (n = 3), viewpoint articles (n = 2), special focus articles (n = 2), survey studies (n = 1), and systematic reviews (n = 1). Additionally, 69% of articles were published in general plastic surgery or aesthetic surgery journals, 13% in facial plastic surgery journals, and 1 study each was published in a craniofacial surgery journal, dermatology journal, endocrinology journal, and medicine journal. A more granular analysis of subject focus revealed that 43% of articles discussed GLP-1 receptor agonist use for the general plastic and aesthetic surgeon, 26% for the facial aesthetic surgeon, 26% for the body contouring surgeon, and only 1 article discussed the medication use in gender-affirming surgery. A summary of the findings and recommendations discussed by each article included in the systematic review is given in Supplemental Table 3. A bias assessment for the cohort studies, cross-sectional studies, and case reports included in this systematic review was conducted and used to determine study quality. As seen in Table 2, all studies that were amenable to this bias and quality assessment were rated as either “good” or “excellent.” The completed bias assessment checklists are summarized in Supplemental Table 4.

**Table 1.** Characteristics of 23 Articles Included in Systematic Review by Year of Publication, Article Type, Journal Focus, and Article Focus, Displayed as Number of Articles and Percentages

	No. of articles (%)
Year of publication	
2017	1 (4)
2023	6 (26)
2024	12 (52)
2025	4 (17)
Article type	
Correspondence and communications	5 (22)
Review	5 (22)
Case report	4 (17)
Retrospective cohort study	3 (13)
Viewpoint	2 (9)
Special focus	2 (9)
Survey study	1 (4)
Systematic review	1 (4)
Journal focus	
Plastic surgery	10 (43)
Aesthetic surgery	6 (26)
Facial plastic surgery	3 (13)
Craniofacial surgery	1 (4)
Dermatology	1 (4)
Other	2 (9)
Article focus	
General plastic/aesthetic surgery	10 (43)
Facial aesthetic surgery	6 (26)
Body contouring surgery	6 (26)
Gender-affirming surgery	1 (4)

**Table 2.** Quality Evaluation for Cohort Studies, Cross-sectional Studies, and Case Reports Included in Systematic Review Based on Bias Assessment Scoring, Displayed as Scores, Percentages, and Overall Rating

	Bias assessment score (%)	Overall rating
Systematic reviews		
Shridharani and Kohan^12^	22/28 (79)	Good
Cohort studies		
Lewis et al^13^	18/20 (90)	Good
Liang et al^14^	16/20 (80)	Good
Toms et al^15^	16/20 (80)	Good
Cross-sectional studies		
Han et al^16^	13/16 (81)	Good
Cohort reports		
de Oliveira Ciaramicolo et al^17^	7/8 (88)	Good
Sleiwah et al^18^	8/8 (100)	Excellent
Taormina et al^19^	8/8 (100)	Excellent
Taraschi and Salgarello^20^	8/8 (100)	Excellent

A thematic analysis of the articles included in this systematic review reveals many recurrent concepts and recommendations.

1. There are several injectable drugs available on the market that are used on- and off-label for weight loss.

Semaglutide (marketed as Ozempic and Wegovy) and liraglutide (marketed as Saxenda) are GLP-1 receptor agonists that impact multiple organ systems, promoting weight loss and significantly slowing gastric emptying. A similar drug named tirzepatide (marketed as Mounjaro and Zepbound) activates both GLP-1 and glucose-dependent insulinotropic polypeptide, increasing insulin sensitivity and early satiety in addition to its favorable effects on weight loss.^21^ The US FDA currently approves Wegovy, Saxenda, and Zepbound for use in chronic weight management. On the other hand, Ozempic and Mounjaro are FDA approved only for patients with Type 2 diabetes and using them for weight loss is characterized as an off-label use of these medications.^22,23^ Some providers argue that the off-label use of Ozempic for achieving rapid weight reduction and meeting BMI goals should be considered for transgender patients given known barriers to pursuing lifestyle changes in this population, frequently delaying access to gender-affirming care.^19^ The potential for off-label use of these medications is further exemplified by a survey study of members of The Aesthetic Society, with one-third of respondents reporting personal use of a GLP-1 receptor agonist (of which two-thirds indicated it was for cosmetic weight loss) and two-thirds of respondents recommending the implementation of medication-assisted weight loss to other plastic surgeons to profit their practice.^16^

1. GLP-1 receptor agonists cause changes to the skin and fat that can contribute to signs of advanced aging.

Patients who undergo moderate medication-assisted weight loss typically lose no more than 25% of their total body weight, so they do not fall into the category of true massive-weight-loss patients. They generally have fewer comorbidities associated with significant weight loss but still experience some of the same issues as massive-weight-loss patients.^24^ This can include changes to facial morphology by reducing the size of the lips, cheeks, and chin, as well as cellular changes resulting in decreased levels of collagen and elastin.^25-27^ Fat loss in the temporal region, cheeks, tear trough, jawline, marionette lines, and nasolabial folds can make patients appear gaunt and resemble advanced facial aging. These effects are more pronounced in older individuals with naturally lower levels of collagen. Evidence to suggest that GLP-1 receptor agonists preferentially result in facial fat atrophy is lacking. Some theorize that GLP-1 receptor agonists merely emphasize the age-related gradual decrease in elastin turnover by accentuating sagging skin under a thinner bed of adipose tissue, rather than preferentially resulting in targeted facial fat atrophy.^28^

Rapid nutrient depletion during weight loss may cause malnutrition. A reduction in fatty acids can weaken the skin barrier, leading to dryness and lack of shine. Patients with significant weight loss may look up to 5 years older than peers without such weight changes.^29^ During weight regain, fat distribution rarely returns to its pre-weight-loss state given established lifestyle modifications and patients’ desire to control their weight as much as possible, potentially contributing to lasting signs of facial aging.^30^ According to 1 correspondence, online searches for “Ozempic® face” are rising and at times even surpassing searches for “Ozempic® side effects,” highlighting the need for physicians across the country to recognize this unintended side effect of anti-obesity medications.^31^ Notably, although considerations about facial fat loss and accelerated aging are important, these are not mentioned in the Ozempic prescribing information.^32^

Other than its effect on facial morphology, semaglutide can also lead to similar changes in breast, body, and buttock morphology, popularized in the media by terms such as “Ozempic® body” and “Ozempic® butt.” According to 1 study, weight loss from GLP-1 receptor agonists administered with a sodium-glucose cotransporter 2 (SGLT-2) inhibitor was primarily because of a reduction in adipose tissue rather than lean tissue as seen on MRI.^33^ These medications may hasten and intensify common issues like postpartum breast deflation and the formation of a ptotic pannus.^21^ Much like the massive-weight-loss community, these patients may seek help from plastic surgeons because of excess skin that can interfere with exercise, intimacy, social interactions, and finding properly fitting clothing.

1. Surgical and nonsurgical options are available for management of “Ozempic® face” and “Ozempic® body.”

Before prescribing Ozempic for weight loss, patients should be counseled about its potential impact on fat distribution. Patients should consider the pros and cons of using Ozempic based on their personal goals and expectations. Treatment for “Ozempic® face” depends on the condition's severity and patient preferences. One option is to stop using Ozempic and switch to a different weight management method that might not have as strong of an effect on facial fat loss. However, this may not be ideal for patients who have experienced significant weight loss and cardiovascular or metabolic improvements with Ozempic, as discontinuation could lead to rebound weight gain. In such cases, injectable dermal fillers can restore facial volume and elasticity. The most common fillers are made of hyaluronic acid, with other options including calcium hydroxyapatite, poly-L-lactic acid, polymethylmethacrylate, and autologous fat transfer.^25^ Additionally, collagen stimulators and skin-tightening techniques (such as radiofrequency microneedling or CO_2_ laser therapy) can restore facial balance.^26,27^ Fillers are generally the primary treatment for addressing volume loss, and physicians should anticipate a growing number of patients seeking these treatments to counteract the effects of “Ozempic® face.”

Surgical options may also help, especially after major weight loss, where more extensive procedures may be needed for optimal results. Changes to facial morphology are best addressed through face and neck lift surgeries, because patients often have more excess skin compared with those who have not lost weight. If needed, extensive skin undermining can be performed to reconcile the excess skin with the superficial musculoaponeurotic system. Patients may present with prominent platysma bands amenable to platysmaplasty. Facial fat grafting is often a useful complementary procedure to address facial volume loss, which typically requires more volume than what is needed for standard facial rejuvenation.^24^ For excess skin and laxity of the breast, abdomen, and extremities, various body contouring procedures, such as mastopexy, panniculectomy, abdominoplasty, brachioplasty, and thighplasty, can significantly help improve symptomatology and patient well-being.^14,15^ Unlike massive-weight-loss patients after bariatric surgery, patients with medication-assisted weight loss have generally experienced less significant weight changes, resulting in better soft tissue quality and a greater likelihood of being candidates for combined procedures.^24,34^

1. The gastrointestinal side effects of GLP-1 receptor agonists help guide perioperative management for patients wishing to undergo elective surgery.

The most commonly reported side effects of GLP-1 receptor agonists are gastrointestinal in nature, including diarrhea, constipation, indigestion, nausea, vomiting, and abdominal pain. These issues are particularly concerning for patients wishing to undergo surgery, as delayed gastric emptying may raise the risk of vomiting and aspiration during intubation. Various holding protocols have been described to mitigate the increased risk for aspiration seen with these medications.^12,21,24,35-37^

The consequences of delayed gastric emptying and reduced gastrointestinal motility may elevate the risks associated with general anesthesia. As a result, the American Society of Anesthesiologists (ASA) has issued consensus-based guidelines for patients using these medications.^38^ Before surgery, the risk of delayed gastric emptying and aspiration should be evaluated by considering drug dosage, titration phase, frequency of dosing, and the presence of gastrointestinal symptoms or other medical comorbidities that may delay gastric emptying. For patients determined to be at elevated risk, a shared decision-making model should be employed, with the option to hold daily medications the day of surgery and weekly medications 1 week before.^38^ For diabetic patients, it is recommended to consult an endocrinologist to avoid hyperglycemia, particularly if the medication is paused longer than recommended. A liquid diet for the 24 h leading up to surgery can be employed. On the day of surgery, if gastrointestinal symptoms are present, the potential risk of regurgitation and pulmonary aspiration should be discussed with the patient, and consideration should be given to delaying the procedure.^38^ If no gastrointestinal symptoms are present and medications were appropriately withheld, it is recommended to proceed with surgery. Older ASA guidelines recommended using “full stomach” precautions or delaying the procedure if medications were not appropriately held before surgery, but the 2025 ASA guidelines leave this decision making up to the physician and patient.

These gastrointestinal side effects, combined with rapid weight loss, are also hypothesized to affect nutrient absorption and increase the risk of malnutrition. In a large multicenter retrospective cohort study, the administration of semaglutide for >6 months preoperatively was associated with many complications associated with a poor nutritional status, including wound dehiscence, delayed wound healing, surgical-site infection, gastrointestinal symptoms (nausea, vomiting, and diarrhea), and prolonged surgical pain.^13^ Because of this, many providers recommend obtaining a thorough nutritional assessment and focusing on nutritional optimization preoperatively, such as with increased protein intake.^13,20,21,39^

1. There are few but important contraindications to the use of GLP-1 receptor agonists.

GLP-1 agonists have been linked to a potential risk of medullary thyroid carcinoma in preclinical studies, which has not been validated in human studies. Given these associations, the administration of GLP-1 receptor agonists is contraindicated in patients with a personal or family history of medullary thyroid carcinoma or multiple endocrine neoplasia syndrome type 2 (MEN 2).^17,36,39,40^ Additionally, caution should be used in patients with previous hypersensitivity to GLP-1 receptor agonists, and the location and severity of symptoms should be assessed before product administration.^12^ Although a potential link between this medication class and pancreatitis as well as pancreatic cancer has been suggested, no clinical trials to date have confirmed this association.^41^ Attention should be paid to patients taking multiple newer antidiabetic medications, such as GLP-1 receptor agonists taken in combination with SGLT-2 inhibitors, because they are at greater risk of experiencing medication adverse effects potentially requiring hospitalization.^12,18^

### Google Trends Search

**Figure 2.**
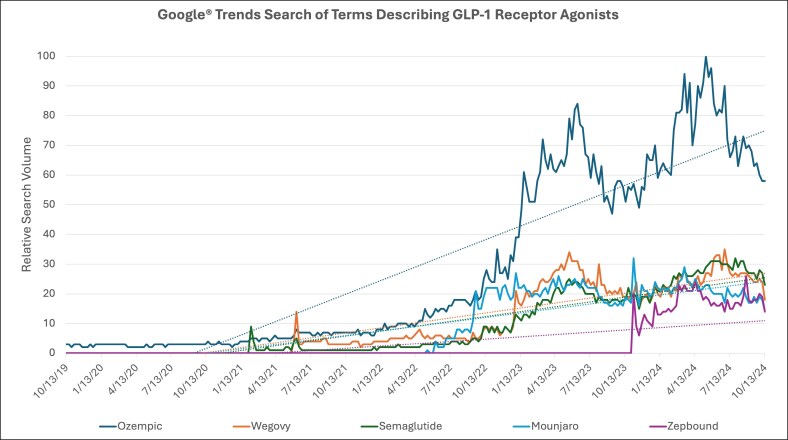
Google Trends search on anti-obesity medications using search terms “Ozempic®,” “Wegovy®,” “Semaglutide,” “Mounjaro®,” and “Zepbound®,” from October 2019 to October 2024. *X*-axis represents time and *Y*-axis represents relative search volume, ranging from insufficient search data (0) to peak popularity (100). Solid lines represent the various search terms (as indicated by color). Dotted lines represent the rate of change for each associated search term (as indicated by color).

Three separate Google Trends searches were conducted. In the first, the search terms compared were “semaglutide,” “Ozempic®,” “Wegovy®,” “Mounjaro®,” and “Zepbound®” (Figure 2). All keywords were also searched on Google News to provide insight into the trends observed through the Google Trends analysis. With a rate of change of +0.018, “semaglutide” had a consistent increase in searches since February 2021. “Ozempic®” similarly had a continuous increase in searches over the timeframe searched, with peaks in April 2024, May 2024, and July 2024. Breakout-related queries, characterized by a rapid surge in search volume compared with previous time periods and used as an indicator for associated emerging trends, included “Ozempic® face,” “Ozempic® celebrities,” and “Ozempic® shortage.” The rate of change for “Ozempic®” was the highest of the search terms in this category at +0.050. With a rate of change of +0.018, “Wegovy®” had a steady increase in searches since June 2021 and a recent peak in May 2023. Breakout-related queries included “Wegovy® shortage.” The rate of change for “Mounjaro®” was similar to “Wegovy®” at +0.017. The trend for “Mounjaro®” has been steadily increasing since May 2022. Recent peaks included November 2023 and March 2024. “Mounjaro® shortage” was similarly a breakout-related query. Lastly, “Zepbound®,” with a rate of change of +0.0085, had seen searches since November 2023 and a recent peak in April 2024. It also had a breakout-related query of “Zepbound® shortage.”

**Figure 3.**
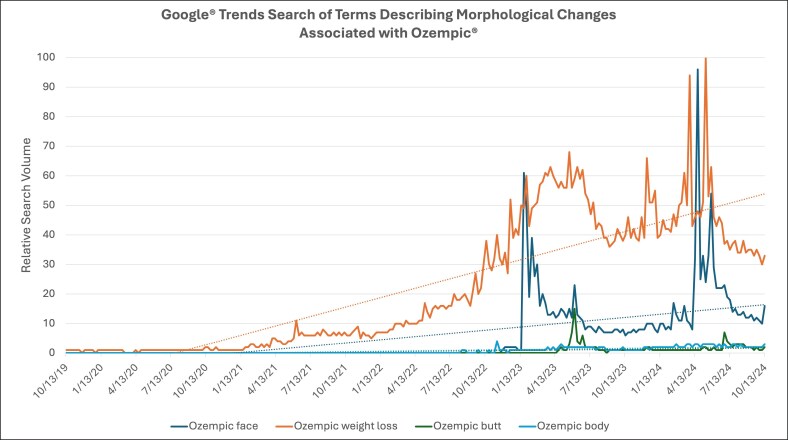
Google Trends search on Ozempic changes to the face and body using search terms “Ozempic® face,” “Ozempic® weight loss,” “Ozempic® butt,” and “Ozempic® body,” from October 2019 to October 2024. *X*-axis represents time and *Y*-axis represents relative search volume, ranging from insufficient search data (0) to peak popularity (100). Solid lines represent the various search terms (as indicated by color). Dotted lines represent the rate of change for each associated search term (as indicated by color).

In the second Google Trends search, the keywords used were: “Ozempic® weight loss,” “Ozempic® face,” “Ozempic® butt,” and “Ozempic® body” (Figure 3). Similarly to “semaglutide,” “Ozempic® weight loss” had a steady increase in searches since February 2021. With a rate of change of +0.035, it peaked in March 2024 and in May 2024. Public searches for “Ozempic® face” have risen since January 2023, with searches recently peaking in April 2024. Breakout-related queries included “plastic surgeons Ozempic® face” and “Ozempic® face celebrities.” The rate of change for “Ozempic® face” was the highest of the search terms in this category at +0.012. Both with a rate of change of +0.0015, “Ozempic® butt” and “Ozempic® body” have not had high search volumes over time. Breakout-related queries for these keywords included “Ozempic® face” and “Ozempic® finger.”

**Figure 4.**
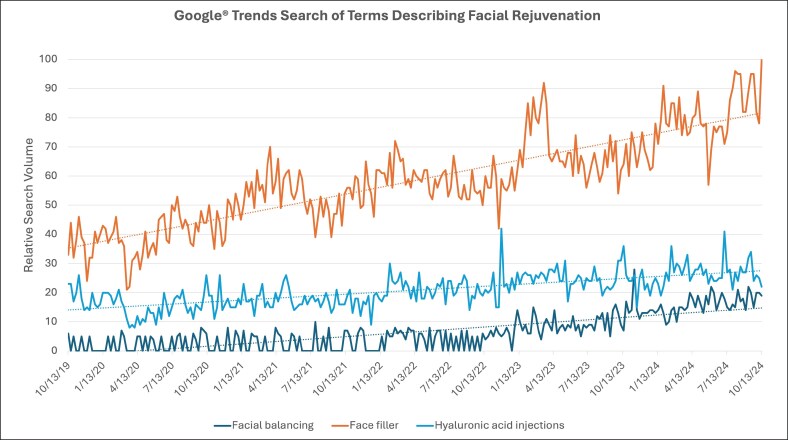
Google Trends search on types of facial rejuvenation using search terms “Facial balancing,” “Face filler,” and “Hyaluronic acid injections,” from October 2019 to October 2024. *X*-axis represents time and *Y*-axis represents relative search volume, ranging from insufficient search data (0) to peak popularity (100). Solid lines represent the various search terms (as indicated by color). Dotted lines represent the rate of change for each associated search term (as indicated by color).

In the final Google® Trends search, the keywords used were: “facial balancing,” “face filler,” and “hyaluronic acid injections” (Figure 4). “Facial balancing” had a steady increase in search volume over time with a rate of change of +0.0090. “Face filler” had a similar growth in searches over time, with a rate of change of +0.025. Searches peaked in February 2023 and April 2024, with “Ozempic® face” being one of the breakout-related queries. Finally, searches for “hyaluronic acid injections” saw a slow increase over time with a rate of change +0.0074 and no notable related queries.

## DISCUSSION

### Systematic Review of the Literature and Recommended Clinical Practice Guidelines

In our systematic review, we reveal that there are many benefits to the use of GLP-1 receptor agonists in plastic surgery. Beyond the cardiovascular improvements, these medications have the potential to aid in preoperative weight optimization, which is particularly relevant in elective plastic surgery, where BMI aids in determining surgical candidacy. Multiple promising cellular and animal studies have explored the effect of GLP-1 receptor agonists on increased angiogenesis, neuroprotection, pain reduction, antioxidant effects, improved wound healing, and decreased inflammation.^34,36^ Human studies have shown decreased postoperative infection rates and benefits in the treatment of polycystic ovarian syndrome and fatty liver disease, with some providers advocating for the use of GLP-1 receptor agonists in autologous breast reconstruction given improved blood sugar and weight control.^34,36^ In one retrospective cohort study, the authors found that patients taking GLP-1 receptor agonists had similar emergency department visits, hospital readmission, and complication rates after body contouring surgery compared with controls.^14^

Despite these benefits, prescribing providers must remain well versed on potential adverse effects and downstream consequences of taking these medications. When assessing weight-loss patients for elective plastic surgery, surgeons should consider the mechanisms and outcomes of their weight loss, as well as factors such as the patient's BMI, weight changes, stability, smoking status, and social support. It is important to screen for the current or future use of injectable weight-loss medications. Patients with high BMI who are considering starting these medications should be counseled not only on their associated weight loss and cardiovascular benefits but also on their side-effect profile—particularly, as it pertains to greater aspiration risk from delayed gastric emptying. The ASA clinical practice guidelines for the safe use of GLP-1 receptor agonists in the perioperative period represent an area that is rapidly changing as new data are shared and providers become more familiar with using this class of medications. We recommend remaining up to date with the anesthesiology literature on this topic, as new guidelines might continue to be published in the near future. Ideally, patients should have at least 6 months of stable weight before surgery, knowing that peak weight loss occurs at 52 weeks after starting GLP-1 receptor agonists.^42^ Discussions should address the risk of weight regain if the medication is discontinued as well as the possibility of future surgery if weight loss continues. Attention should be paid to possible signs of malnutrition that could impact wound healing, and preoperative diet should be optimized with increased protein intake. A postoperative plan for continuing or stopping weight-loss drugs should be established, considering the higher risk of ileus for those using opioid pain medication.

With a growing awareness of the accelerated fat loss caused by this medication class resulting in features of “Ozempic® face” and “Ozempic® body,” it is paramount for prescribing providers, whether they be primary care physicians or plastic surgeons, to counsel patients on these potential morphological changes. As we have discussed, the accelerated evidence of facial aging associated with GLP-1 receptor agonist use is likely a direct consequence of rapid weight loss resulting in preferential volume loss of adipose tissue without compensatory collagen remodeling at the level of the skin. Moreover, we currently lack evidence to suggest that GLP-1 receptor agonists preferentially result in fat atrophy of the face. For these reasons, the term “Ozempic® face,” which was created and popularized by news outlets, likely represents a transient trend rather than new medical terminology or a new side effect associated with this medication class. Moreover, because medication discontinuation results in weight regain of up to two-thirds a patient's original weight, it is possible that the gaunt appearance characteristic of “Ozempic® face” might become less prominent over time as the initial surge in prescriptions stabilizes and patients discontinue use of these medications.

Fortunately, the plastic surgeon is already well equipped to manage changes to the morphology of the face and body associated with weight-loss medications. The loss of facial volume seen with “Ozempic® face” can be addressed with dermal fillers or fat grafting to the temples, tear troughs, cheeks, and nasolabial folds. Sunken eyes with significantly ptotic skin can be addressed with a lower blepharoplasty, including skin resection. Globally loose skin with a longer, aged-appearing face will require skin excision through a lower facelift to address skin redundancy. Neurotoxin injection and laser therapy for skin tightening can be employed to address more prominent facial wrinkles. Finally, skin ptosis with loss of elasticity from rapid adipose tissue loss in the breast, body, and extremities can be addressed with various body contouring procedures. As seen by this systematic review, there is a growing body of literature to educate the plastic surgery community on this topic. The onus should in turn be placed on us to educate our associated and referring specialties, so that any provider responsible for prescribing and managing this medication class can provide patients with appropriate counseling on the development of characteristic morphological changes. We also recommend discussing with patients that if and when GLP-1 receptor agonists are interrupted, weight regain may occur, including facial weight gain, and dissolution of any injected filler might be pursued to avoid significant facial fullness and the appearance of increased facial weight.

### Google Trends Analysis and Economic Implications

Our Google Trends analysis reveals that public searches for anti-obesity medications, including semaglutide products Ozempic and Wegovy, as well as tirzepatide products Mounjaro and Zepbound, have been consistently rising over time. “Semaglutide” searches have been rising since February 2021, when the landmark randomized controlled trial on weight loss associated with subcutaneous semaglutide injections was published.^4^ Similarly, searches for “Wegovy®,” “Mounjaro®,” and “Zepbound®” have been consistently rising since the dates each of these medications garnered FDA approval.^23^ With the help of Google News, we were able to link search peaks to relevant current events, such as “Ozempic® face” gaining popularity in plastic surgery blogs, various celebrities announcing their use of these medications for weight loss, and medication shortages when supply was limited by manufacturers.^43-48^ Public searches for “Ozempic® face” have consistently risen since January 2023, when the first article mentioning “Ozempic® face” was reported in the news.^49^ Search volumes for “Ozempic® butt” and “Ozempic® body” have remained low over time, with corresponding fewer news outlets reporting on these body changes.^50^ Searches for “facial balancing,” referring to nonsurgical procedures to restore harmony and symmetry to the face, have been steadily increasing over time, whereas searches for “face filler” peaked at the same time “Ozempic® face” was widely reported in the news.^43,49^

Rise in the popularity of “Ozempic® face” has been linked to rising searches for “face filler” and “plastic surgeons.” This indicates that as the unintended morphological changes of GLP-1 receptor agonists become increasingly reported, so does the interest in seeking procedures aimed at facial rejuvenation. With the rise of newer anti-obesity medications such as Mounjaro, it becomes exceedingly important to continue to report on and be aware of potential side effects associated with these medications. The plastic surgery community can prepare for the rising needs of the patient population taking weight-loss medications by understanding their mechanism of action and side-effect profile, recognizing the physical changes attributed to their use and how these can be addressed surgically and cosmetically to tailor treatment to each individual patient, and finally establishing clear perioperative guidelines as discussed above.

Sales of anti-obesity medications have significantly increased in recent years. Novo Nordisk, the pharmaceutical company responsible for producing many GLP-1 receptor agonist medications, including Ozempic, Wegovy, and Saxenda, reported an annual profit equivalent to over $12 billion in 2023.^51^ Ozempic contributed significantly to this revenue, accounting for 41% of total sales in 2023 and totaling nearly $14 billion.^51^ Mounjaro is expected to outperform Ozempic as the top-selling drug in the obesity and diabetes markets, with projected sales of $27 billion by 2029.^52^ The popularization of the term “Ozempic® face” in media outlets exemplifies this. Many companies are capitalizing on this trend by selling products targeting the unfavorable weight loss associated with anti-obesity medications, such as the food and beverage company Nestlé (Vevey, Switzerland) developing dietary supplements intended to counter the effects of “Ozempic® face” or the skincare company Galderma (Zug, Switzerland) claiming its collagen treatments and fillers can help reverse the appearance of “Ozempic® face.”^53,54^ Coining the term “Ozempic® face” has not only empowered patients to recognize what it is and how to address it, but it has also started to empower large companies to enter the market of unintended weight loss “reversal.” With the use of GLP-1 receptor agonists growing at an unprecedented rate, it becomes paramount for medical providers to remain well-informed on any updates to the risk profile of this medication class.

### Future Directions

There was a notable lack of prospective or patient-reported outcome studies in our systematic review, because the topic of morphological face and body changes associated with GLP-1 receptor agonists has only recently been garnering scientific and public attention. Exploring the psychosocial and quality-of-life impact of advanced facial aging resulting from GLP-1 receptor agonist administration, particularly in a more vulnerable patient population such as those with elevated BMI, associated comorbidities, or body dysmorphia, is of paramount importance. Future longitudinal studies on the long-term effects of GLP-1 receptor agonists on facial structure and studies discussing the implementation of specific interventions to address these changes are needed to address this clinical knowledge gap.

### Limitations

Several limitations should be considered when interpreting the results of this systematic review. Of the studies included, only 3 were cohort studies that compared GLP-1 receptor agonist administration with a control group. Other than these studies, 1 survey study of aesthetic plastic surgeons and 1 systematic review; all other articles represented lower quality evidence in the form of case reports, opinion pieces, and reviews. Limitations of our study include the potential for bias in the studies reviewed, heterogeneous populations, dosage and administration differences for the various medications reviewed, and limited data on long-term use of these medications, with only 1 study published before the year 2023. As with all systematic reviews, our results should be interpreted within the context of the demographic characteristics of the articles reviewed.

## CONCLUSIONS

Several weight-loss medications are available on the market that plastic surgeons should be aware of. Many studies support the beneficial cellular and structural changes associated with GLP-1 receptor agonist administration. Despite these benefits, prescribing providers must remain well versed on potential adverse effects and downstream consequences of taking this class of medications, including gastrointestinal side effects that help guide perioperative management for patients wishing to undergo elective surgery, and changes to the skin and fat that can contribute to signs of advanced aging. Public searches for “Ozempic® face” have been linked to coincidently rising searches for “face filler” and “plastic surgeons,” suggesting that people are more likely to seek rejuvenation procedures as these changes become popularized. Many companies are also starting to capitalize on the rise of “Ozempic® face” by selling products meant to counter these effects. The rapidly rising production and sales for this class of medications warrants our continued vigilance in an effort to maximize patient safety and satisfaction. The plastic surgery community can prepare for the rising needs of the population taking these medications by understanding their mechanism, side-effect profile, recognizing the physical changes attributed to their use, and how these can be addressed surgically and cosmetically.

## Supplementary Material

ojaf056_Supplementary_Data
