# Supplementary material for: “Ozempic Face” in Plastic Surgery: A Systematic Review of the Literature on GLP-1 Receptor Agonist Mediated Weight Loss and Analysis of Public Perceptions
Source: Aesthet Surg J Open Forum. 2025 Jun 11;7:ojaf056. doi: 10.1093/asjof/ojaf056 (PMC12232544; doi:10.1093/asjof/ojaf056)
Supplement: ojaf056_Supplementary_Data [file ojaf056_supplementary_data.zip › Supp Table 3- Article descriptions.docx]

| **Article identifier** | **Title** | **Publication journal** | **Article type** | **Summary of findings and/or recommendations** |
| --- | --- | --- | --- | --- |
| Borab et al. (2024) | Emerging Role of Semaglutide and GLP-1 Agonist Medications in Plastic Surgery: A Note of Caution | Plast Reconstr Surg | Special topic | Review of the benefits of GLP-1 receptor agonists and their use in plastic surgery. Note of caution on aspiration risk. Importance of preoperative discussions (weight loss peaking at 52 weeks, no surgery during active weight loss, potential weight regain after medication cessation). Provides summary of available GLP-1 receptor agonists, mode and timing of administration, and FDA approved indications. |
| Capla et al. (2024) | Patient Evaluation and Surgical Staging | Clin Plast Surg | Review | Practice paradigm for patient evaluation, physical examination, surgical planning, and staging of body contouring procedures for patients with massive weight loss and medication-assisted weight loss. Highlights importance of thorough history and physical and involvement of anesthesia team in surgical planning, including limiting combination procedures to <6 hours. |
| de Oliveira Ciaramicolo et al. (2024) | Late Facial Edema After Lip Filling With Hyaluronic Acid: Possible Association With the Use of Ozempic | J Craniofac Surg | Case report | Report of severe nodular facial edema 3 months after hyaluronic acid filler injection worsened by initiation of semaglutide and resolved with hyaluronidase injection and semaglutide discontinuation. |
| Han et al. (2024) | Practice Patterns and Perspectives of the Off-Label Use of GLP-1 Agonists for Cosmetic Weight Loss | Aesthet Surg J | Survey study | Survey of 368 members of The Aesthetic Society on the use and management of GLP-1 receptor agonists, revealing that most respondents use this medication class for cosmetic weight loss, either as part of a weight loss center, a wellness spa, or in preparation for body contouring surgery. More than half of respondents believe GLP-1 receptor agonists are profitable for business, and even more so recommend their use to other plastic surgeons. Discrepancies in responses included differences in preoperative risk assessment (one quarter of respondents do not screen  for high-risk conditions considered to be contraindications). Most respondents use compounding pharmacies to prescribe GLP-1 receptor agonists, even if the practice is discouraged by the FDA. Most respondents believe GLP-1 receptor agonists are an effective long-term weight loss solution, even if studies suggest rebound weight gain after discontinuation. |
| Hansen et al. (2023) | Letter to the Editor: Comment on Implications of Ozempic and Other Semaglutide Medications for Facial Plastic  Surgeons | Facial Plast Surg | Correspondence and communications | Commentary on the article by Humphrey et al. (2023), noting that Ozempic is not approved by the FDA for chronic weight management, that facial fat loss is not listed in the Ozempic prescribing information, and that there are many cardiovascular benefits to Ozempic-mediated weight loss that should not be overlooked. |
| Haykal et al. (2024) | The Role of GLP-1 Agonists in Esthetic Medicine: Exploring the Impact of Semaglutide on Body Contouring and Skin Health | J Cosmet Dermatol | Review | Commentary reviewing the aesthetic consequences of weight loss caused by semaglutide administration and offering practice paradigms for surgical and non-surgical management. Questions raised about variable impact of semaglutide on superficial vs deep fat compartments, effects on other soft tissues, and timing of aesthetic interventions relative to each patient’s weight loss journey. |
| Humphrey et al. (2023) | Corrigendum: Implications of Ozempic and Other Semaglutide Medications for Facial Plastic Surgeons | Facial Plast Surg | Review | Review of the pharmacologic effects of Ozempic on weight loss and gastrointestinal side effects, including risk of malnutrition. Review of the aging effects of Ozempic on the face, including suggested surgical and non-surgical options for management. |
| Lewis et al. (2024) | Semaglutide and Postoperative Outcomes in Non-Diabetic Patients Following Body Contouring Surgery | Aesthet Surg J | Retrospective cohort study | Multicenter analysis of 109,142 patients examining the relationship between preoperative semaglutide and postoperative complications in nondiabetic patients after body contouring surgery. Preoperative semaglutide for >6 months was associated with higher rates of all complications, including wound dehiscence, surgical site infection, nausea/vomiting/diarrhea, surgical pain, and hypertrophic scarring. Authors advocate for operative staging and preoperative nutritional assessment. |
| Liang et al. (2025) | Postoperative Outcomes in  Body Contouring Procedures Following GLP-1 Receptor Agonist Use: A 10-Year  Analysis | Aesthet Surg J | Retrospective cohort study | Postoperative evaluation of 590 patients undergoing non-breast body contouring surgery at a single institution. Comparison of GLP-1 receptor agonist group and control group revealed similar ED visits, hospital readmission, and complication rates between the groups—except lower cellulitis incidence in GLP-1 receptor agonist group. |
| Mansour et al. (2024) | The rise of "Ozempic Face": Analyzing trends and treatment challenges associated with rapid facial weight loss induced by GLP-1 agonists | J Plast Reconstr Aesthet Surg | Correspondence and communications | Google Trends analysis of various reported Ozempic side effects, including “Ozempic face”, to raise awareness of the latter as an unintended side effect of weight loss medications. |
| Oleru et al. (2024) | Surgical Implications of GLP-1 Agonists for Weight Loss: Are These Patients Suitable for Elective Surgery? | Plast Reconstr Surg | Viewpoint | Opinion piece questioning the safety of performing elective surgery on patients with elevated BMIs. Offers institutional peri-operative guidelines for patients taking GLP-1 receptor agonists, including medication cessation for two full cycles, clear liquid diet for 24 hours before surgery, and NPO at  midnight the evening before surgery. |
| O’Neill et al. (2024) | Injectable Weight Loss Medications in Plastic Surgery: What We Know,  Perioperative Considerations, and Recommendations for the Future | Plast Reconstr Surg Glob Open | Special topic | Overview of the history, risks, and peri-operative considerations of GLP-1 receptor agonists. Overview of the morphologic effects of these medications as well as the impact to other surgical specialties. Practice recommendations are provided, including the importance of a thorough history and physical, adherence to American Society of Anesthesiologists guidelines, nutritional optimization, and weight stability prior to surgery. |
| Paranzino et al. (2025) | Medical Weight Management  Considerations in Plastic Surgery | Plast Reconstr Surg | Review | Overview of indications for anti-obesity therapy and history of anti-obesity medications. Summary of GLP-1 receptor agonists including benefits, limitations, comparison with bariatric surgery, propensity to regain weight after medication discontinuation, and concern for nutritional deficiencies. Peri-operative recommendations and practice guidelines for the plastic surgeon are provided. |
| Ragsdale et al. (2024) | Plastic surgery amid a semaglutide epidemic: Direction for future studies and service implications | J Plast Reconstr Aesthet Surg | Correspondence and communications | Brief review of promising cellular, animal, and human studies on the benefits of GLP-1 receptor agonists on angiogenesis, inflammation, and wound healing. Authors advocate for increase in plastic surgery residency body contouring minimums to account for rising patient population taking weight loss medications. |
| Sciscent et al. (2024) | Semaglutide, Popularly Known as Ozempic-What the Facial Plastic Surgeon Needs to Know | Facial Plast Surg Aesthet Med | Viewpoint | Opinion piece counseling plastic surgeons that “Ozempic face” is not a side effect unique to semaglutide administration, but rather refers to rapid weight loss changes affecting the face. |
| Shridharani et al. (2023) | The Emerging Role of Injectable Weight Loss  Medications in Plastic Surgery: A Systematic Review | Aesthet Surg J | Systematic review | Systematic review on the safety and efficacy of SGLT-2 inhibitors and GLP-1 receptor agonists, used alone or in combination. Sixteen RCTs reviewed including 10,492 patients. Average weight loss with GLP-1 receptor agonists was 2-4 kg, increased to 3-5 kg if co-administered with SGLT-2 inhibitors. Most common adverse effect of GLP-1 receptor agonists was mild to moderate dose-related GI symptoms. Co-administration of dapagliflozin and exenatide resulted in significant weight loss in non-diabetic patients but was associated with adverse events in all participants, including two hospitalizations. Review importance of thorough history and physical and adherence to the American Society of Anesthesiologists preoperative fasting guidelines. |
| Sleiwah et al. (2017) | Euglycaemic ketoacidosis: a potential new hazard to plastic surgery day case and inpatient procedures | BMJ Case Rep | Case report | Report of patient undergoing elective abdominoplasty with bilateral mastopexy on concurrent GLP-1 receptor agonist and SGLT-2 inhibitor (liraglutide and empagliflozin, held day of surgery and resumed day after) developing severe diabetic ketoacidosis with euglycemia, a rare but serious complication of newer antidiabetic medications. |
| Stanton et al. (2025) | Glucagon-Like Peptide-1 Agonists: A Practical Overview for Plastic and Reconstructive  Surgeons | Ann Plast Surg | Review | Overview of the history, development, mode and timing of administration, and side effect profile of the various available GLP-1 receptor agonists. Summary of existing non-plastic surgery literature suggesting that the greatest glycemic control is achieved with high-dose semaglutide (2 mg) and tirzepatide (15 mg). Overview of diverse possible applications of GLP-1 receptor agonists, including treatment of polycystic ovarian syndrome and fatty liver disease in humans; neuroprotective, pain reduction, antioxidant, and wound healing benefits in preclinical studies. Authors advocate for use of GLP-1 receptor agonists in autologous breast reconstruction, given improved blood sugar and weight control. Also, potential benefit in reducing lymphedema and improving limb salvage in critical limb ischemia. Provides recommendation to restart GLP-1 receptor agonists at the lowest dose postoperatively with a long titration protocol. |
| Taormina et al. (2023) | Meeting the Body Mass Index Requirement for Gender-affirming Surgery Using Antiobesity Medication | JCEM Case Rep | Case report | Report of transgender female seeking bilateral breast augmentation not meeting BMI cutoff (<35) and with limitations to pursuing lifestyle modifications due to body dysmorphia and fear of discrimination. BMI cutoff achieved in 3 months with 14% total weight reduction after initiation and slow up-titration of semaglutide. Authors advocate for use of GLP-1 receptor agonists in transgender patients given significant delays in achieving BMI cutoff for gender affirming surgery. |
| Taraschi et al. (2025) | GLP-1 Agonists in Plastic Surgery: Impact on Aesthetic Outcomes-Two Case Reports | Aesthetic Plast Surg | Case report | Report of two patients undergoing elective breast surgery (implant exchange with mastopexy and reduction mammoplasty) while taking GLP-1 receptor agonists (liraglutide, continued peri-operatively by one patient and discontinued by the other) who developed delayed wound healing with fat necrosis postoperatively. |
| Tay (2023) | A new challenge for facial plastic surgeons | J Plast Reconstr Aesthet Surg | Correspondence and communications | Warning for facial plastic surgeons on the effect of Ozempic on facial fat redistribution, including recommended dermal filler injection sites for patients experiencing “Ozempic face”. |
| Thompson et al. (2023) | Peri-operative management of Ozempic and other GLP-1 agonists during cosmetic surgery: What plastic surgeons need to know | J Plast Reconstr Aesthet Surg | Correspondence and communications | Recommendation to adhere to the American Society of Anesthesiologists guidelines on peri-operative cessation of GLP-1 receptor agonists, with the potential for longer cessation periods if warranted by the risk/benefit analysis on maintaining weight control. |
| Toms et al. (2024) | Prevalence Patterns of Body Contouring Procedures Among Injectable Glucagon-like Peptide-1 Receptor Agonist Users | Aesthet Surg J | Retrospective cohort study | Multicenter analysis of 5,595 patients examining the relationship between GLP-1 receptor agonists and body contouring surgery. Found a significant dose-dependent correlation between Ozempic, Wegovy, and liraglutide use and various body contouring procedures. Also found a higher mean surgery count and decreased time to surgery compared to post-bariatric surgery patients for users of all medications. |
