# Supplementary material for: “Ozempic Face” in Plastic Surgery: A Systematic Review of the Literature on GLP-1 Receptor Agonist Mediated Weight Loss and Analysis of Public Perceptions
Source: Aesthet Surg J Open Forum. 2025 Jun 11;7:ojaf056. doi: 10.1093/asjof/ojaf056 (PMC12232544; doi:10.1093/asjof/ojaf056)
Supplement: ojaf056_Supplementary_Data [file ojaf056_supplementary_data.zip › Supp Table 4- Bias assessment checklists.docx]

**Han et al. (2024)**

| **Item** | **Criteria** | **Answers** |
| --- | --- | --- |
| 1 | *Is the hypothesis/aim/objective of the study clearly described?* | Yes = 1 |
| 2 | *Are the main outcomes to be measured clearly described in the Introduction or Methods section?* | Yes = 1 |
| 3 | *Are the characteristics of the patients included in the study clearly described?* | Yes = 1 |
| 4 | *Are the interventions of interest clearly described?* | Yes = 1 |
| 5 | *Are the distributions of principal confounders in each group of subjects to be compared clearly described?* | No = 0 |
| 6 | *Are the main findings of the study clearly described?* | Yes = 1 |
| 7 | *Does the study provide estimates of the random variability in the data for the main outcomes?* | Yes = 1 |
| 8 | *Have all important adverse events that may be a consequence of the intervention been reported?* | No = 0 |
| 9 | *Have the characteristics of patients lost to follow-up been described?* | Not scored for cross-sectional studies |
| 10 | *Have actual probability values been reported for the main outcomes except where the probability value is less than 0.001?* | Yes = 1 |
| 11 | *Were the subjects asked to participate in the study representative of the entire population from which they were recruited?* | Yes = 1 |
| 12 | *Were those subjects who were prepared to participate representative of the entire population from which they were recruited?* | Yes = 1 |
| 13 | *Were the staff, places, and facilities where the patients were treated, representative of the treatment the majority of patients receive?* | Yes = 1 |
| 14 | *Was an attempt made to blind study subjects to the intervention they have received?* | Not scored for cross-sectional studies |
| 15 | *Was an attempt made to blind those measuring the main outcomes of the intervention?* | Not scored for cross-sectional studies |
| 16 | *If any of the results of the study were based on “data dredging”, was this made clear?* | Yes = 1 |
| 17 | *In trials and cohort studies, do the analyses adjust for different lengths of follow-up of patients, or in case-control studies, is the time period between the intervention and outcome the same for cases and controls?* | Not scored for cross-sectional studies |
| 18 | *Were the statistical tests used to assess the main outcomes appropriate?* | Yes = 1 |
| 19 | *Was compliance with the intervention/s reliable?* | Not scored for cross-sectional studies |
| 20 | *Were the main outcome measures used accurate (valid and reliable)?* | Yes = 1 |
| 21 | *Were the patients in different intervention groups (trials and cohort studies) or were the cases and controls (case-control studies) recruited from the same population?* | Not scored for cross-sectional studies |
| 22 | *Were study subjects in different intervention groups (trials and cohort studies) or were the cases and controls (case-control studies) recruited over the same period of time?* | Not scored for cross-sectional studies |
| 23 | *Were study subjects randomized to intervention groups?* | Not scored for cross-sectional studies |
| 24 | *Was the randomized intervention assignment concealed from both patients and health care staff until recruitment was complete and irrevocable?* | Not scored for cross-sectional studies |
| 25 | *Was there adequate adjustment for confounding in the analyses from which the main findings were drawn?* | Not scored for cross-sectional studies |
| 26 | *Were losses of patients to follow-up taken into account?* | Not scored for cross-sectional studies |
| 27 | *Was a power analysis performed?* | Not scored for cross-sectional studies |
| **Total score = 13/16** | | |

**Lewis et al. (2024)**

| **Item** | **Criteria** | **Answers** |
| --- | --- | --- |
| 1 | *Is the hypothesis/aim/objective of the study clearly described?* | Yes = 1 |
| 2 | *Are the main outcomes to be measured clearly described in the Introduction or Methods section?* | Yes = 1 |
| 3 | *Are the characteristics of the patients included in the study clearly described?* | Yes = 1 |
| 4 | *Are the interventions of interest clearly described?* | Yes = 1 |
| 5 | *Are the distributions of principal confounders in each group of subjects to be compared clearly described?* | Yes = 2 |
| 6 | *Are the main findings of the study clearly described?* | Yes = 1 |
| 7 | *Does the study provide estimates of the random variability in the data for the main outcomes?* | Yes = 1 |
| 8 | *Have all important adverse events that may be a consequence of the intervention been reported?* | Yes = 1 |
| 9 | *Have the characteristics of patients lost to follow-up been described?* | No = 1 |
| 10 | *Have actual probability values been reported for the main outcomes except where the probability value is less than 0.001?* | Yes = 1 |
| 11 | *Were the subjects asked to participate in the study representative of the entire population from which they were recruited?* | Yes = 1 |
| 12 | *Were those subjects who were prepared to participate representative of the entire population from which they were recruited?* | Yes = 1 |
| 13 | *Were the staff, places, and facilities where the patients were treated, representative of the treatment the majority of patients receive?* | Yes = 1 |
| 14 | *Was an attempt made to blind study subjects to the intervention they have received?* | Not scored for uncontrolled studies |
| 15 | *Was an attempt made to blind those measuring the main outcomes of the intervention?* | Not scored for uncontrolled studies |
| 16 | *If any of the results of the study were based on “data dredging”, was this made clear?* | Yes = 1 |
| 17 | *In trials and cohort studies, do the analyses adjust for different lengths of follow-up of patients, or in case-control studies, is the time period between the intervention and outcome the same for cases and controls?* | Yes = 1 |
| 18 | *Were the statistical tests used to assess the main outcomes appropriate?* | Yes = 1 |
| 19 | *Was compliance with the intervention/s reliable?* | Yes = 1 |
| 20 | *Were the main outcome measures used accurate (valid and reliable)?* | Yes = 1 |
| 21 | *Were the patients in different intervention groups (trials and cohort studies) or were the cases and controls (case-control studies) recruited from the same population?* | Not scored for uncontrolled studies |
| 22 | *Were study subjects in different intervention groups (trials and cohort studies) or were the cases and controls (case-control studies) recruited over the same period of time?* | Not scored for uncontrolled studies |
| 23 | *Were study subjects randomized to intervention groups?* | Not scored for uncontrolled studies |
| 24 | *Was the randomized intervention assignment concealed from both patients and health care staff until recruitment was complete and irrevocable?* | Not scored for uncontrolled studies |
| 25 | *Was there adequate adjustment for confounding in the analyses from which the main findings were drawn?* | Not scored for uncontrolled studies |
| 26 | *Were losses of patients to follow-up taken into account?* | Unable to determine = 0 |
| 27 | *Was a power analysis performed?* | Not scored for uncontrolled studies |
| **Total score = 18/20** | | |

**Liang et al. (2025)**

| **Item** | **Criteria** | **Answers** |
| --- | --- | --- |
| 1 | *Is the hypothesis/aim/objective of the study clearly described?* | Yes = 1 |
| 2 | *Are the main outcomes to be measured clearly described in the Introduction or Methods section?* | Yes = 1 |
| 3 | *Are the characteristics of the patients included in the study clearly described?* | Yes = 1 |
| 4 | *Are the interventions of interest clearly described?* | Yes = 1 |
| 5 | *Are the distributions of principal confounders in each group of subjects to be compared clearly described?* | No = 0 |
| 6 | *Are the main findings of the study clearly described?* | Yes = 1 |
| 7 | *Does the study provide estimates of the random variability in the data for the main outcomes?* | Yes = 1 |
| 8 | *Have all important adverse events that may be a consequence of the intervention been reported?* | Yes = 1 |
| 9 | *Have the characteristics of patients lost to follow-up been described?* | No = 0 |
| 10 | *Have actual probability values been reported for the main outcomes except where the probability value is less than 0.001?* | Yes = 1 |
| 11 | *Were the subjects asked to participate in the study representative of the entire population from which they were recruited?* | Yes = 1 |
| 12 | *Were those subjects who were prepared to participate representative of the entire population from which they were recruited?* | Yes = 1 |
| 13 | *Were the staff, places, and facilities where the patients were treated, representative of the treatment the majority of patients receive?* | Yes = 1 |
| 14 | *Was an attempt made to blind study subjects to the intervention they have received?* | Not scored for uncontrolled studies |
| 15 | *Was an attempt made to blind those measuring the main outcomes of the intervention?* | Not scored for uncontrolled studies |
| 16 | *If any of the results of the study were based on “data dredging”, was this made clear?* | Yes = 1 |
| 17 | *In trials and cohort studies, do the analyses adjust for different lengths of follow-up of patients, or in case-control studies, is the time period between the intervention and outcome the same for cases and controls?* | Yes = 1 |
| 18 | *Were the statistical tests used to assess the main outcomes appropriate?* | Yes = 1 |
| 19 | *Was compliance with the intervention/s reliable?* | Yes = 1 |
| 20 | *Were the main outcome measures used accurate (valid and reliable)?* | Yes = 1 |
| 21 | *Were the patients in different intervention groups (trials and cohort studies) or were the cases and controls (case-control studies) recruited from the same population?* | Not scored for uncontrolled studies |
| 22 | *Were study subjects in different intervention groups (trials and cohort studies) or were the cases and controls (case-control studies) recruited over the same period of time?* | Not scored for uncontrolled studies |
| 23 | *Were study subjects randomized to intervention groups?* | Not scored for uncontrolled studies |
| 24 | *Was the randomized intervention assignment concealed from both patients and health care staff until recruitment was complete and irrevocable?* | Not scored for uncontrolled studies |
| 25 | *Was there adequate adjustment for confounding in the analyses from which the main findings were drawn?* | Not scored for uncontrolled studies |
| 26 | *Were losses of patients to follow-up taken into account?* | Unable to determine = 0 |
| 27 | *Was a power analysis performed?* | Not scored for uncontrolled studies |
| **Total score = 16/20** | | |

**Shridharani et al. (2023)**

| **Item** | **Criteria** | **Answers** |
| --- | --- | --- |
| 1 | *Is the hypothesis/aim/objective of the study clearly described?* | Yes = 1 |
| 2 | *Are the main outcomes to be measured clearly described in the Introduction or Methods section?* | Yes = 1 |
| 3 | *Are the characteristics of the patients included in the study clearly described?* | Yes = 1 |
| 4 | *Are the interventions of interest clearly described?* | Yes = 1 |
| 5 | *Are the distributions of principal confounders in each group of subjects to be compared clearly described?* | No = 0 |
| 6 | *Are the main findings of the study clearly described?* | Yes = 1 |
| 7 | *Does the study provide estimates of the random variability in the data for the main outcomes?* | Yes = 1 |
| 8 | *Have all important adverse events that may be a consequence of the intervention been reported?* | Yes = 1 |
| 9 | *Have the characteristics of patients lost to follow-up been described?* | No = 0 |
| 10 | *Have actual probability values been reported for the main outcomes except where the probability value is less than 0.001?* | Yes = 1 |
| 11 | *Were the subjects asked to participate in the study representative of the entire population from which they were recruited?* | Yes = 1 |
| 12 | *Were those subjects who were prepared to participate representative of the entire population from which they were recruited?* | Yes = 1 |
| 13 | *Were the staff, places, and facilities where the patients were treated, representative of the treatment the majority of patients receive?* | Yes = 1 |
| 14 | *Was an attempt made to blind study subjects to the intervention they have received?* | Yes = 1 |
| 15 | *Was an attempt made to blind those measuring the main outcomes of the intervention?* | Yes = 1 |
| 16 | *If any of the results of the study were based on “data dredging”, was this made clear?* | Yes = 1 |
| 17 | *In trials and cohort studies, do the analyses adjust for different lengths of follow-up of patients, or in case-control studies, is the time period between the intervention and outcome the same for cases and controls?* | Yes = 1 |
| 18 | *Were the statistical tests used to assess the main outcomes appropriate?* | Yes = 1 |
| 19 | *Was compliance with the intervention/s reliable?* | Yes = 1 |
| 20 | *Were the main outcome measures used accurate (valid and reliable)?* | Yes = 1 |
| 21 | *Were the patients in different intervention groups (trials and cohort studies) or were the cases and controls (case-control studies) recruited from the same population?* | Yes = 1 |
| 22 | *Were study subjects in different intervention groups (trials and cohort studies) or were the cases and controls (case-control studies) recruited over the same period of time?* | Yes = 1 |
| 23 | *Were study subjects randomized to intervention groups?* | Yes = 1 |
| 24 | *Was the randomized intervention assignment concealed from both patients and health care staff until recruitment was complete and irrevocable?* | Yes = 1 |
| 25 | *Was there adequate adjustment for confounding in the analyses from which the main findings were drawn?* | Unable to determine = 0 |
| 26 | *Were losses of patients to follow-up taken into account?* | Unable to determine = 0 |
| 27 | *Was a power analysis performed?* | No = 0 |
| **Total score = 22/28** | | |

**Toms et al. (2024)**

| **Item** | **Criteria** | **Answers** |
| --- | --- | --- |
| 1 | *Is the hypothesis/aim/objective of the study clearly described?* | Yes = 1 |
| 2 | *Are the main outcomes to be measured clearly described in the Introduction or Methods section?* | Yes = 1 |
| 3 | *Are the characteristics of the patients included in the study clearly described?* | Yes = 1 |
| 4 | *Are the interventions of interest clearly described?* | Yes = 1 |
| 5 | *Are the distributions of principal confounders in each group of subjects to be compared clearly described?* | No = 0 |
| 6 | *Are the main findings of the study clearly described?* | Yes = 1 |
| 7 | *Does the study provide estimates of the random variability in the data for the main outcomes?* | Yes = 1 |
| 8 | *Have all important adverse events that may be a consequence of the intervention been reported?* | Yes = 1 |
| 9 | *Have the characteristics of patients lost to follow-up been described?* | No = 0 |
| 10 | *Have actual probability values been reported for the main outcomes except where the probability value is less than 0.001?* | Yes = 1 |
| 11 | *Were the subjects asked to participate in the study representative of the entire population from which they were recruited?* | Yes = 1 |
| 12 | *Were those subjects who were prepared to participate representative of the entire population from which they were recruited?* | Yes = 1 |
| 13 | *Were the staff, places, and facilities where the patients were treated, representative of the treatment the majority of patients receive?* | Yes = 1 |
| 14 | *Was an attempt made to blind study subjects to the intervention they have received?* | Not scored for uncontrolled studies |
| 15 | *Was an attempt made to blind those measuring the main outcomes of the intervention?* | Not scored for uncontrolled studies |
| 16 | *If any of the results of the study were based on “data dredging”, was this made clear?* | Yes = 1 |
| 17 | *In trials and cohort studies, do the analyses adjust for different lengths of follow-up of patients, or in case-control studies, is the time period between the intervention and outcome the same for cases and controls?* | Yes = 1 |
| 18 | *Were the statistical tests used to assess the main outcomes appropriate?* | Yes = 1 |
| 19 | *Was compliance with the intervention/s reliable?* | Yes = 1 |
| 20 | *Were the main outcome measures used accurate (valid and reliable)?* | Yes = 1 |
| 21 | *Were the patients in different intervention groups (trials and cohort studies) or were the cases and controls (case-control studies) recruited from the same population?* | Not scored for uncontrolled studies |
| 22 | *Were study subjects in different intervention groups (trials and cohort studies) or were the cases and controls (case-control studies) recruited over the same period of time?* | Not scored for uncontrolled studies |
| 23 | *Were study subjects randomized to intervention groups?* | Not scored for uncontrolled studies |
| 24 | *Was the randomized intervention assignment concealed from both patients and health care staff until recruitment was complete and irrevocable?* | Not scored for uncontrolled studies |
| 25 | *Was there adequate adjustment for confounding in the analyses from which the main findings were drawn?* | Not scored for uncontrolled studies |
| 26 | *Were losses of patients to follow-up taken into account?* | Unable to determine = 0 |
| 27 | *Was a power analysis performed?* | Not scored for uncontrolled studies |
| **Total score = 16/20** | | |

De Oliveira Ciaramicolo et al. (2024)

| **Item** | **Criteria** | **Answers** |
| --- | --- | --- |
| 1 | Were patient’s demographic characteristics clearly described? | Yes = 1 |
| 2 | Was the patient’s history clearly described and presented as a timeline? | Yes = 1 |
| 3 | Was the current clinical condition of the patient on presentation clearly described? | Yes = 1 |
| 4 | Were diagnostic tests or assessment methods and the results clearly described? | No = 0 |
| 5 | Was the intervention(s) or treatment procedure(s) clearly described? | Yes = 1 |
| 6 | Was the post-intervention clinical condition clearly described? | Yes = 1 |
| 7 | Were adverse events (harms) or unanticipated events identified and described? | Yes = 1 |
| 8 | Does the case report provide takeaway lessons? | Yes = 1 |
| **Total score = 7/8** | | |

Sleiwah et al. (2017)

| **Item** | **Criteria** | **Answers** |
| --- | --- | --- |
| 1 | Were patient’s demographic characteristics clearly described? | Yes = 1 |
| 2 | Was the patient’s history clearly described and presented as a timeline? | Yes = 1 |
| 3 | Was the current clinical condition of the patient on presentation clearly described? | Yes = 1 |
| 4 | Were diagnostic tests or assessment methods and the results clearly described? | Yes = 1 |
| 5 | Was the intervention(s) or treatment procedure(s) clearly described? | Yes = 1 |
| 6 | Was the post-intervention clinical condition clearly described? | Yes = 1 |
| 7 | Were adverse events (harms) or unanticipated events identified and described? | Yes = 1 |
| 8 | Does the case report provide takeaway lessons? | Yes = 1 |
| **Total score = 8/8** | | |

Taormina et al. (2023)

| **Item** | **Criteria** | **Answers** |
| --- | --- | --- |
| 1 | Were patient’s demographic characteristics clearly described? | Yes = 1 |
| 2 | Was the patient’s history clearly described and presented as a timeline? | Yes = 1 |
| 3 | Was the current clinical condition of the patient on presentation clearly described? | Yes = 1 |
| 4 | Were diagnostic tests or assessment methods and the results clearly described? | Yes = 1 |
| 5 | Was the intervention(s) or treatment procedure(s) clearly described? | Yes = 1 |
| 6 | Was the post-intervention clinical condition clearly described? | Yes = 1 |
| 7 | Were adverse events (harms) or unanticipated events identified and described? | Yes = 1 |
| 8 | Does the case report provide takeaway lessons? | Yes = 1 |
| **Total score = 8/8** | | |

Taraschi et al. (2025)

| **Item** | **Criteria** | **Answers** |
| --- | --- | --- |
| 1 | Were patient’s demographic characteristics clearly described? | Yes = 1 |
| 2 | Was the patient’s history clearly described and presented as a timeline? | Yes = 1 |
| 3 | Was the current clinical condition of the patient on presentation clearly described? | Yes = 1 |
| 4 | Were diagnostic tests or assessment methods and the results clearly described? | Yes = 1 |
| 5 | Was the intervention(s) or treatment procedure(s) clearly described? | Yes = 1 |
| 6 | Was the post-intervention clinical condition clearly described? | Yes = 1 |
| 7 | Were adverse events (harms) or unanticipated events identified and described? | Yes = 1 |
| 8 | Does the case report provide takeaway lessons? | Yes = 1 |
| **Total score = 8/8** | | |
