# Supplementary figures and images for: “Ozempic Face” in Plastic Surgery: A Systematic Review of the Literature on GLP-1 Receptor Agonist Mediated Weight Loss and Analysis of Public Perceptions
Source: Aesthet Surg J Open Forum. 2025 Jun 11;7:ojaf056. doi: 10.1093/asjof/ojaf056 (PMC12232544; doi:10.1093/asjof/ojaf056)

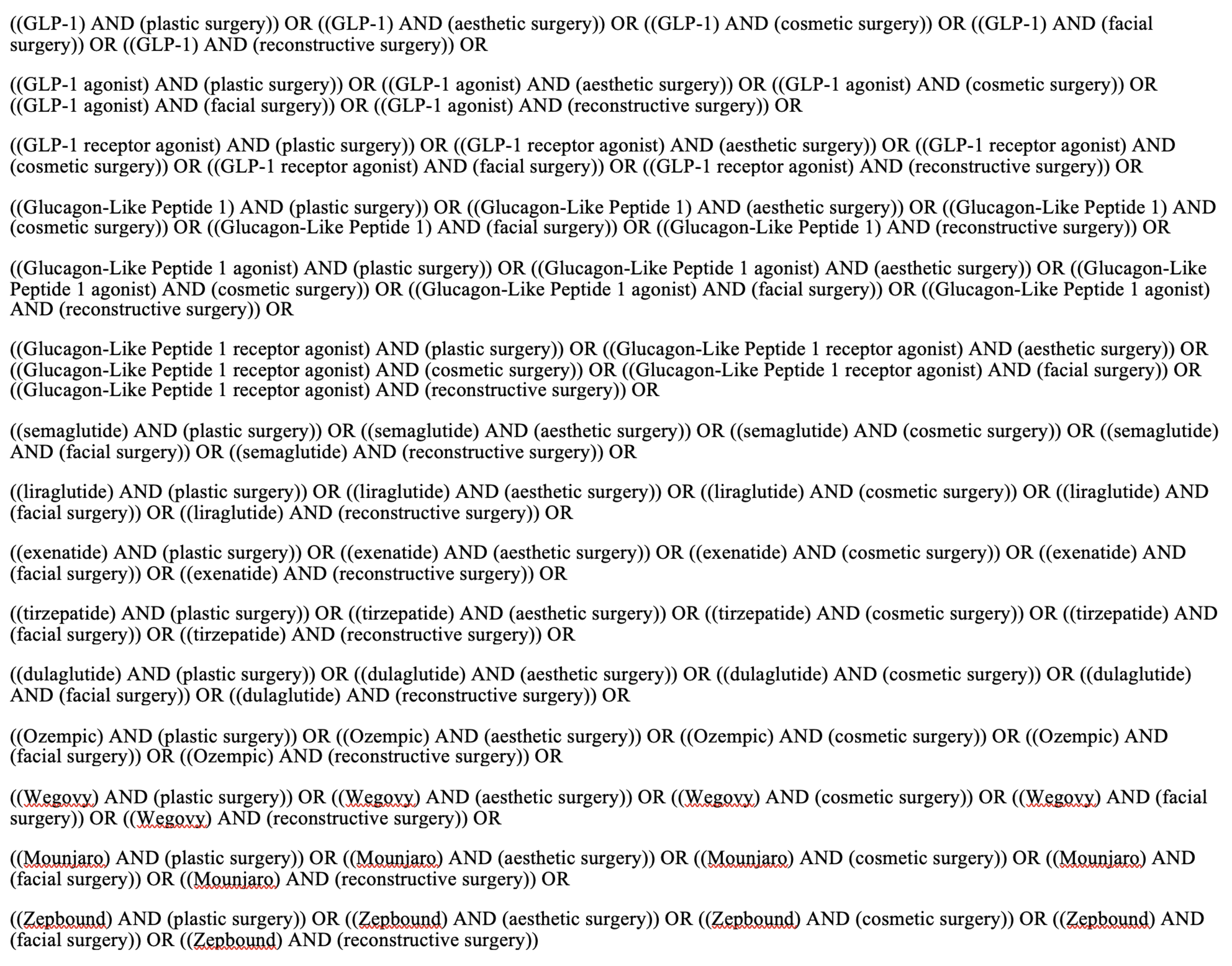

Supplement: ojaf056_Supplementary_Data [file ojaf056_supplementary_data.zip › Supp Fig 1- PubMed search terms.tiff]
